# Shear Deformation Dissipates Energy in Biofilaments

**DOI:** 10.1038/s41598-018-29905-6

**Published:** 2018-08-03

**Authors:** Ameneh Maghsoodi, Noel Perkins

**Affiliations:** Department of Mechanical Engineering, University of Michigan, Ann Arbor, MI 48109 USA

## Abstract

Thermally fluctuating biofilaments possessing porous structures or viscoelastic properties exhibit energy losses from internal friction as well as external friction from drag. Prior models for internal friction account for energy dissipation solely from the dynamic bending of filaments. In this paper, we present a new energy dissipation model that captures the important effects of dynamic shear in addition to bending. Importantly, we highlight that shear-induced friction plays a major role in energy dissipation for shorter filaments and for shorter wavelengths (larger wavenumbers). The new model exhibits coupled shear-bending energy relaxation on two distinct time scales in lieu of a single time scale predicted by bending alone. We employ this model to interpret results from prior experiments on the internal friction of thermally fluctuating chromosomes and the drag-induced friction of thermally fluctuating microtubules. The examples confirm the energy relaxation on two time scales associated with internal friction and on two length scales associated with external friction. Overall, this new model that accounts for shear deformation yields superior estimates of energy dissipation for fluctuating biofilaments.

## Introduction

**Figure 1:**
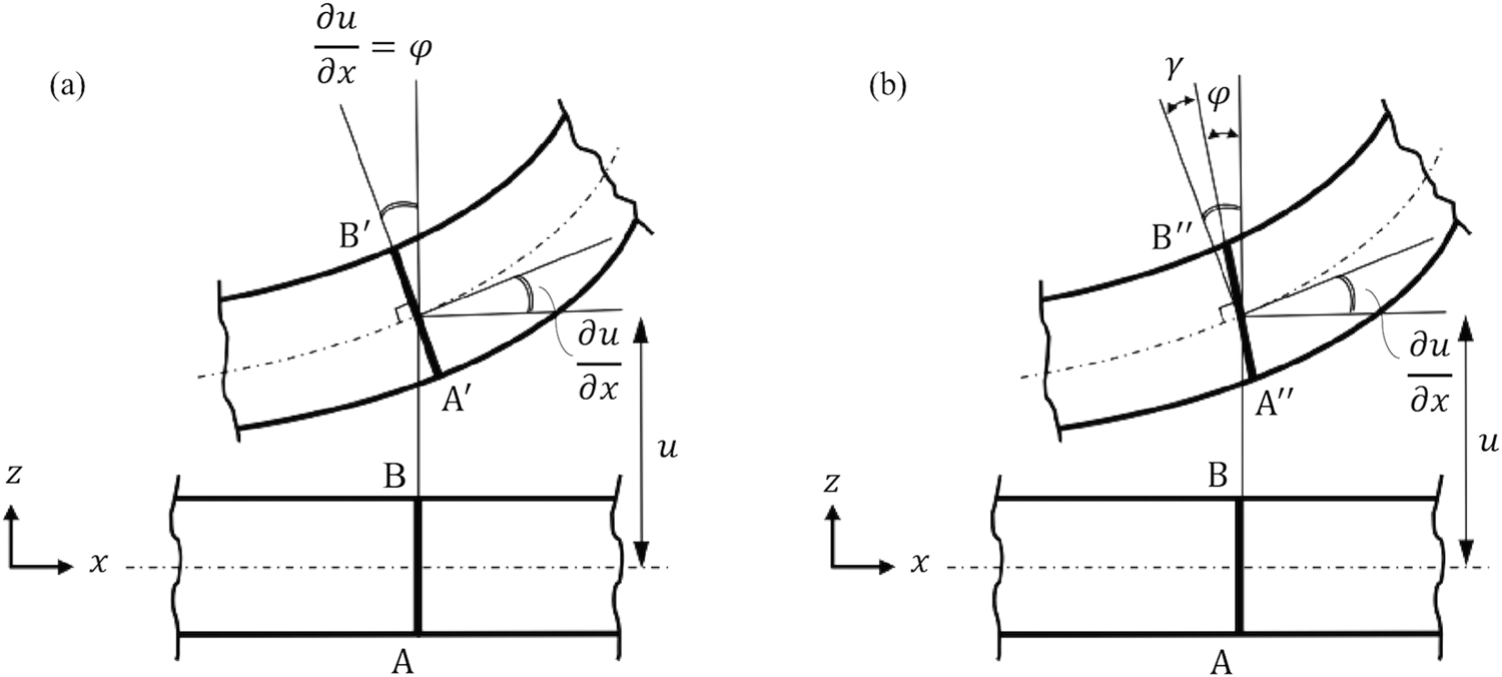
Schematic of rod cross section before deformation (bottom) and after deformation (top). The worm-like chain model employs Euler-Bernoulli theory in which the deformed cross section A′B′ remains perpendicular to the rod centerline (**a**). In Timoshenko theory, the deformed cross section A″B″ does not remain perpendicular to the rod centerline due to the additional rotation due to shear (**b**). In particular, *φ* and *γ* are the rotations due to bending deformation and shear deformation, respectively, and thus $\frac{\partial u}{\partial x}=\varphi +\gamma$ is the total rotation of the deformed cross section.

Biofilaments including microtubules, DNA, and actin filaments are semiflexible micro-scale polymer structures that perform essential functions in living cells. Understanding the dynamical behavior and the material properties of biofilaments are central to understanding their structure-function relations. A variety of models describe the material stiffness and thermal fluctuations of biofilaments^1–6^. Primary among these is the worm-like chain (WLC) model^7,8^ which represents a biofilament as a continuous isotropic rod undergoing dynamic bending while subject to thermal excitation and hydrodynamic drag^9–11^. Poirier and Marko^12^ extend the WLC model to account for internal friction due to dynamic bending. Subsequent studies have employed that model to describe the internal dissipation of biofilaments in addition to external friction due to hydrodynamic drag^4,11–13^. However, the WLC model^8^ and its extension for bending-induced internal friction^12^ are based on classical Euler-Bernoulli theory for beam bending^14^ which tacitly assumes that the cross sections of the filament remain planar and perpendicular to the (fluctuating) filament centerline; see Fig. 1a. These kinematic assumptions, which remain accurate only for long filaments and long wavelength (small wavenumber) fluctuations, limit the applicability of the WLC model.

However, understanding the dynamics of short biofilaments remains of great interest when one considers the many short-range interactions within the cell achieved through biofilaments as well as biophysical experiments on short biofilaments. For example, short (<10 μm long^15^) microtubules are actively recruited in intracellular transport and cellular mitosis while short (<5 μm long^16^) actin filaments, responsible for cell motility, dominate the distribution of actin. Key measures of biofilament length include the ratio of the filament length to diameter *L*/2*r* and the ratio of the wavelength of dynamic fluctuations to the filament radius *λ*/*r*. For short filaments (*L*/2*r* < 10)^14,17^ or short wavelength fluctuations (*λ*/*r* < 10)^14^, the effect of shear deformation becomes important relative to bending deformation. At these length scales, the filament exhibits direct shear deformation and the filament cross sections no longer remain perpendicular to the filament centerline (see Fig. 1b) as assumed in the WLC model (see Fig. 1a). This shear effect in biofilaments can be quite pronounced as exposed herein in the context of internal friction. The effect of shear on the stiffness properties of short biofilaments has previously been observed in^18,19^. For example, Pampaloni *et al*.^19^ measured the persistence length of thermally fluctuating microtubules having lengths from 2.6 to 47.5 μm. Both theory and experiment confirm that the persistence length of short microtubules (shorter than 21 μm) is length-dependent as a consequence of shear deformation. Thus, it stands to reason that shear deformation in thermally fluctuating biofilaments may also significantly affect *dissipation* properties as well. To address this hypothesis, we present a new model for thermally fluctuating biofilaments, based on Timoshenko beam theory, which considers shear deformation as an additional source of elastic deformation and energy dissipation. This model reveals important effects of dynamic shear (above those due to dynamic bending) on both internal and external dissipation mechanisms. Results demonstrate that shear deformation leads to qualitatively new energy dissipation behaviors including dissipation dynamics on two time scales associated with internal friction and on two length scales associated with external friction. These new theoretical predictions successfully describe the experimental trends observable in studies of fluctuating chromosomal fragments^12^ and microtubules^13^.

## Methods

We propose a model of energy dissipation for thermally fluctuating biofilaments possessing short lengths (*L*/2*r* < 10)^14,17^ or short wavelength fluctuations (*λ*/*r* < 10)^14^ in which shear deformation is not negligible. To this end, we employ Timoshenko beam theory^14^ which models the coupled bending-shear deformations of elastic rods. The governing Langevin formulation is

1 $$B\frac{{\partial }^{2}\varphi }{\partial {x}^{2}}+\kappa S(\frac{\partial u}{\partial x}-\varphi )+{\eta ^{\prime} }_{b}I\frac{{\partial }^{3}\varphi }{\partial {x}^{2}\partial t}+{\eta ^{\prime} }_{s}\,A(\frac{{\partial }^{2}u}{\partial x\partial t}-\frac{\partial \varphi }{\partial t})=0$$

2 $$\kappa S(\frac{\partial \varphi }{\partial x}-\frac{{\partial }^{2}u}{\partial {x}^{2}})+{\eta ^{\prime} }_{s}\,A(\frac{{\partial }^{2}\varphi }{\partial x\partial t}-\frac{{\partial }^{3}u}{\partial {x}^{2}\partial t})+\eta \frac{\partial u}{\partial t}=n(x,t)$$

in which *u*(*x*, *t*) denotes the fluctuating transverse displacement of the filament (in directions perpendicular to the filament centerline) due to random thermal excitation *n*(*x*, *t*), *x* denotes the filament contour length coordinate, and *t* denotes time. The quantities *φ* and $\frac{\partial u}{\partial x}-\varphi$ are the components of the rotation of the filament cross section due to bending deformation and shear deformation, respectively. The filament bending stiffness *B* = *EI* is composed of the filament’s Young’s modulus *E* and area moment of inertia *I* while the filament shear stiffness *S* = *GA* is composed of the filament’s shear modulus *G* and cross sectional area *A*. The quantity *κ* denotes the Timoshenko shear correction factor which, for a filament with circular cross section, is *κ* = 0.75^14^. Here, *η* denotes the external hydrodynamic drag coefficient (Stoke’s regime), and ${\eta ^{\prime} }_{b}$ and ${\eta ^{\prime} }_{s}$ are the internal dissipation coefficients due to bending and shear deformations, respectively. Consequently, the third and fourth terms in (1) and the second term in (2) model the internal dissipation due to shear (coefficient ${\eta ^{\prime} }_{s}$) and bending (coefficient ${\eta ^{\prime} }_{b}$). Consistent with the Langevin formulation, the inertial terms that otherwise appear in Timoshenko theory^14^ are neglected. A derivation of (1–2) is included in the Supplemental Materials.

As in prior analyses^12^, energy dissipation can be quantified by computing the autocorrelation function for the transverse displacement *u* for thermal fluctuations assuming ideal (white) thermal noise. To this end, the autocorrelation function ${\mathcal R} (T)$ for *u* follows from a Fourier transform of (1) and (2)

3 $${U}_{q\omega }=\iint u(x,t){{\rm{e}}}^{i(qx-\omega t)}{\rm{d}}x\,{\rm{d}}t$$

4 $${\Phi }_{q\omega }=\iint \varphi (x,t){{\rm{e}}}^{i(qx-\omega t)}{\rm{d}}x\,{\rm{d}}t$$

in which *U*_*qω*_ and *Φ*_*qω*_ denote the (double) Fourier transforms of the transverse displacement *u* and the rotation *φ*, respectively. The quantities *q* and *ω* are the wavenumber and frequency of propagating waves, respectively. The resulting autocorrelation function (derived in the Supplemental Materials) becomes

5 $$ {\mathcal R} (T)={R}_{1}\,{{\rm{e}}}^{\frac{-T}{{\tau }_{1}}}+\,{R}_{2}\,{{\rm{e}}}^{\frac{-T}{{\tau }_{2}}}$$

6 $${\tau }_{1}=\sqrt{\frac{2{\rm{M}}}{{\rm{N}}+\sqrt{{{\rm{N}}}^{2}-4{\rm{MP}}}}},\,{\tau }_{2}=\sqrt{\frac{2{\rm{M}}}{{\rm{N}}-\sqrt{{{\rm{N}}}^{2}-4{\rm{MP}}}}}$$

Here *τ*_1_ and *τ*_2_ are *two* distinct energy relaxation times and *T* is the lag-time. Thus, this result immediately reveals that energy relaxation occurs on the *two* time scales *τ*_1_ and *τ*_2_ that are functions of the wavenumber *q*, internal viscosities ${\eta ^{\prime} }_{b}$ and ${\eta ^{\prime} }_{s}$, and hydrodynamic drag *η* through the quantities M, N, and P detailed in the Supplemental Materials. We discuss these time scales and the effects of shear deformation in detail below.

In the limit of long filaments or long wavelengths, the shear deformation is negligible and the total rotation of the cross section due to bending alone obeys the kinematic constraint $\frac{\partial u}{\partial x}=\varphi .$ Upon employing this constraint, the formulation above recovers the single time scale autocorrelation function for the WLC model (based on Euler-Bernoulli beam theory) employed in^12^.

7 $$ {\mathcal R} (T)=R\,{{\rm{e}}}^{\frac{-T}{\tau }}$$

8 $$\tau =\frac{\eta +{\eta ^{\prime} }_{b}I{q}^{4}}{B{q}^{4}}$$

For further reference, (8) becomes

9 $$\tau =\frac{\eta }{B{q}^{\ast 4}}{L}^{4}+\frac{{\eta ^{\prime} }_{b}I}{B}$$

in which q^*^ = *qL* is a non-dimensional wavenumber with *L* being the filament length.

While the above analysis (5 and 6) reveals the effects of shear on internal friction, one can perform a parallel analysis to expose the effects of shear on external friction due to hydrodynamic drag alone. In this case $({\eta ^{\prime} }_{s}={\eta ^{\prime} }_{b}=0)$, Eqs (1 and 2) simplify to

10 $$B\frac{{\partial }^{2}\varphi }{\partial {x}^{2}}+\kappa S(\frac{\partial u}{\partial x}-\varphi )=0$$

11 $$\kappa S(\frac{\partial \varphi }{\partial x}-\frac{{\partial }^{2}u}{\partial {x}^{2}})+\eta \frac{\partial u}{\partial t}=n(x,t)$$

The associated autocorrelation ${\mathcal R} (T)$ for *u* and its relaxation time *τ*_*d*_ become

12 $$ {\mathcal R} (T)={R}_{d}\,\exp (\frac{-T}{{\tau }_{d}})$$

13 $${\tau }_{d}=\frac{\kappa S\eta +B\eta {q}^{2}}{\kappa SB{q}^{4}}$$

Details of this analysis are provided in the Supplemental Materials. For further reference, (13) is expanded as

14 $${\tau }_{d}=\frac{\eta }{B{q}^{\ast 4}}{L}^{4}+\frac{\eta }{\kappa S{q}^{\ast 2}}{L}^{2}$$

to reveal the explicit dependence of this relaxation time on *two* length scales. By contrast, for long filaments or long wavelengths, the WLC model ((9) with ${\eta ^{\prime} }_{b}=0$) predicts that the relaxation time *τ* due to external friction depends on a single length scale per

15 $$\tau =\frac{\eta }{B{q}^{\ast 4}}{L}^{4}.$$

## Results and Discussion

### Internal friction in large and small wavenumber limits

Equation (5), based on Timoshenko beam theory, explicitly accounts for the direct shear of filaments which is ignored in the prior formulations based on Euler-Bernoulli beam theory. Importantly, (5) reveals that the energy relaxation arises on *two time scales τ*_1_ and *τ*_2_. Thus, the physics of internal friction when shear is included is qualitatively different from that when shear is ignored for which single time scale relaxation (7) occurs.

**Figure 2:**
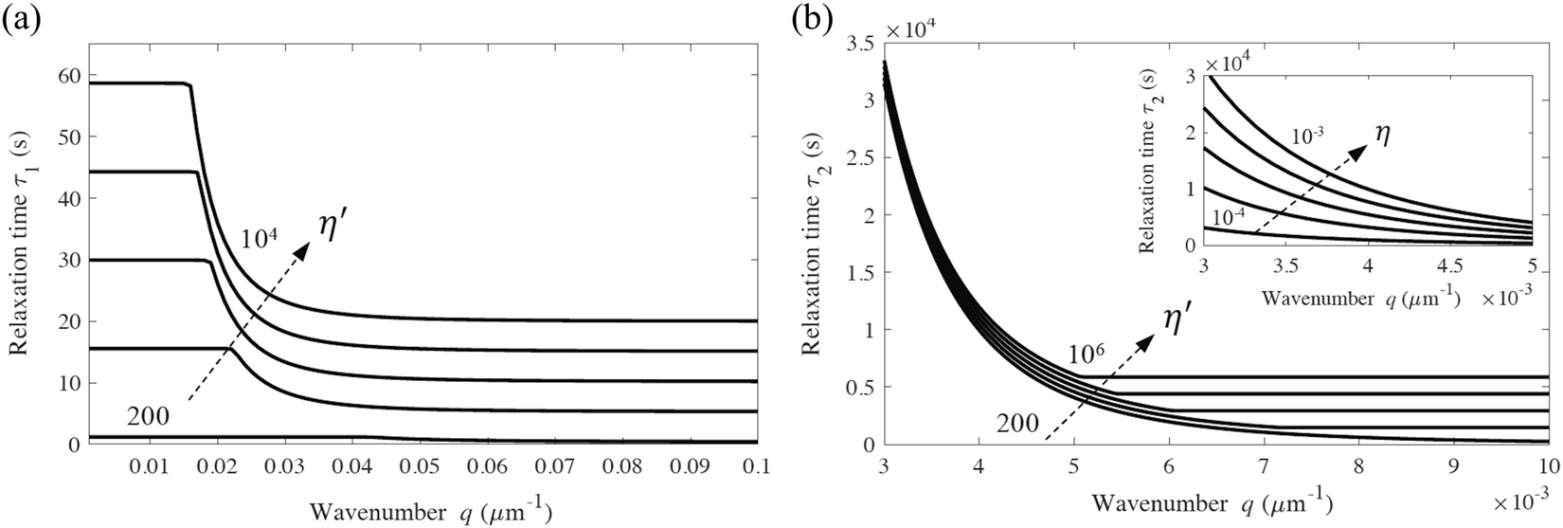
Main figure: Relaxation times (**a**) *τ*_1_ and (**b**) *τ*_2_ for the autocorrelation of transverse displacement of a thermally fluctuating chromosome. Properties: *E* = 500 Pa, *r* = 1 μm, *η* = 0.001 kg/(m.s)^12^, *G* = 227 Pa, and ${\eta ^{\prime} }_{s}={\eta ^{\prime} }_{b}=\eta ^{\prime}$ ranging from 200 to 10^4^ kg/(m.s) for (**a**) and from 200 to 10^6^ kg/(m.s) for (**b**). Inset: In the small wavenumber limit, the relaxation time *τ*_2_ is independent of *η*′, however, it scales with the hydrodynamic drag coefficient, *η*, ranging from 0.0001 to 0.001 kg/(m.s).

Figure 2 illustrates the dependence of the two relaxation times *τ*_1_ and *τ*_2_ (6) with wavenumber *q* over a wide range of values for ${\eta ^{\prime} }_{s}={\eta ^{\prime} }_{b}=\eta ^{\prime}$ as examples. The parameters selected pertain to a thermally fluctuating chromosomal filament^12^ having *E* = 500 Pa, *r* = 1 μm, *η* = 0.001, and *G* = 227 Pa (Poisson’s ratio *υ* = 0.1)^20^. Inspection of Fig. 2 reveals that *τ*_1_ and *τ*_2_ become independent of wavenumber in the large wavenumber (*q* → ∞) limit for which (6) yields

16 $${\tau }_{1}\approx \frac{{\eta ^{\prime} }_{b}I}{B},\,{\tau }_{2}\approx \frac{{\eta ^{\prime} }_{s}A}{\kappa S}$$

For comparison, the relaxation time (8) from Euler-Bernoulli theory reduces to

17 $$\tau \approx \frac{{\eta ^{\prime} }_{b}\,I}{B}$$

From (16) and (17), the relaxation time *τ*_1_ from Timoshenko theory recovers the relaxation time *τ* from Euler-Bernoulli theory, in the large wavenumber limit. However, the relaxation time *τ*_2_ defines a second time scale, a shear relaxation time that cannot be captured by modeling thermal fluctuations using Euler-Bernoulli theory.

Figure 2 also reveals the expected limiting behaviors at small wavenumbers. In the small wavenumber (*q* → 0) limit, (6) yields

18 $${\tau }_{1}\approx \frac{{\eta ^{\prime} }_{s}A}{\kappa S},\,{\tau }_{2}\approx \frac{\eta }{B{q}^{4}}\to \infty $$

Thus, *τ*_2_ reproduces the same limit from Euler-Bernoulli theory (8) at the small wavenumber limit which confirms the overwhelming influence of hydrodynamic drag over bending-induced internal friction^12^. As further illustrated in Fig. 2b in this limit, the relaxation time *τ*_2_ becomes independent of internal dissipation (*η*′), and scales with hydrodynamic drag (*η*); see Fig. 2b (inset). However, *τ*_1_ depends on the shear internal dissipation coefficient ${\eta ^{\prime} }_{s}$ in this limit. In particular, for *q* → 0, the wavelength approaches infinity, and the filament fluctuations reduce to essentially rigid body motions without significant internal friction but with significant external friction due to hydrodynamic drag.

### Experimental evidence of shear effect on internal friction

**Figure 3:**
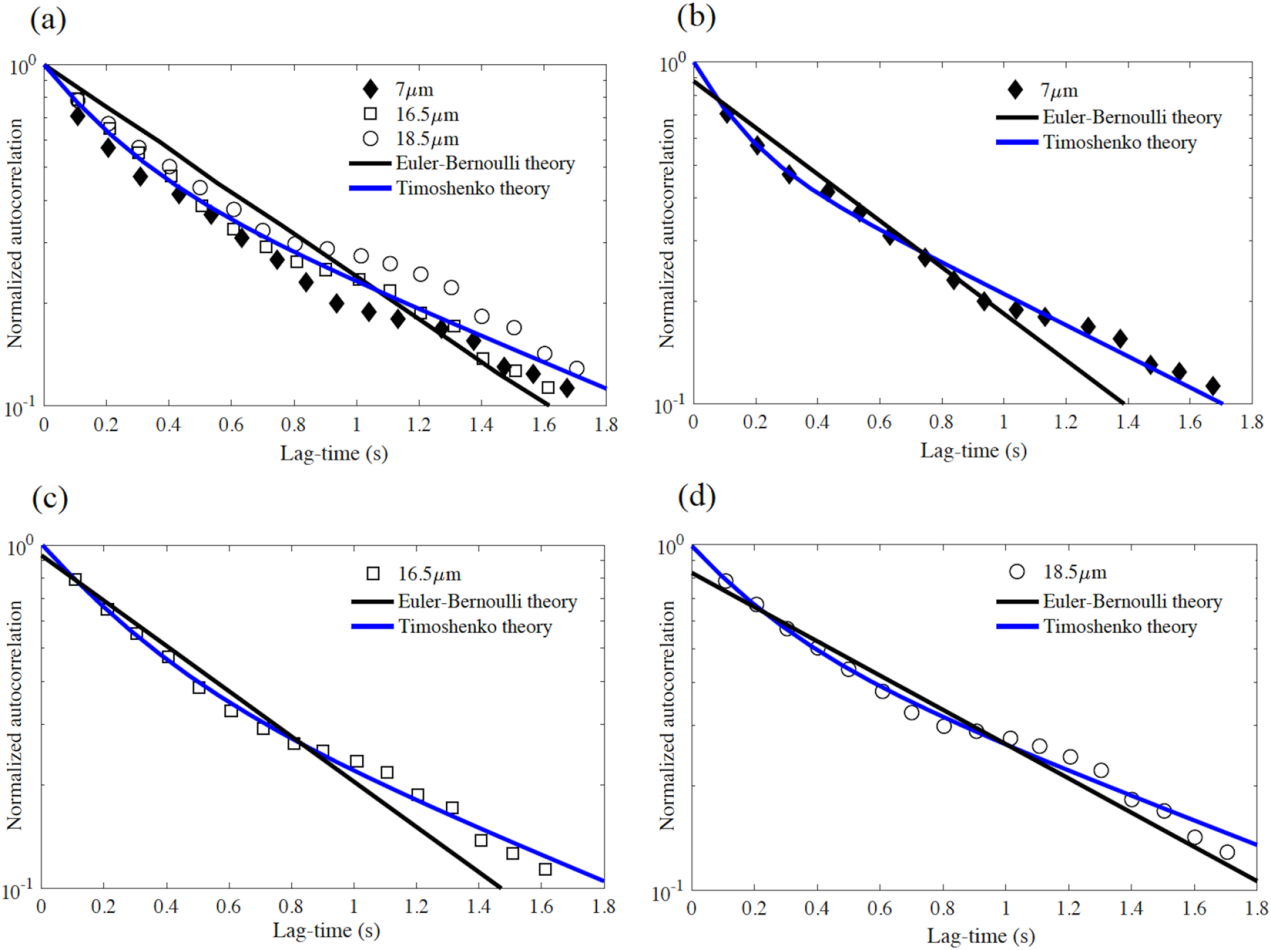
(**a**) Experimental autocorrelations of transverse displacement of thermally fluctuating chromosomes reported in^12^ for all three chromosome lengths and with model fits determined by Timoshenko theory (5) and Euler-Bernoulli theory (7). Autocorrelations for (**b**) 7 μm-chromosome, (**c**) 16.5 μm-chromosome, and (**d**) 18.5 μm-chromosome.

We employ the new model, based on Timoshenko theory, to evaluate results of prior experiments on the internal friction for thermally fluctuating chromosomes of varying length^12^. The chromosomal segments considered formed cantilevers of lengths 7, 16.5, and 18.5 μm having estimated properties: *E* = 500 Pa, *G* = 227 Pa, *r* = 1 μm, and *η* = 0.001 kg/(m.s). The measured autocorrelation of the transverse fluctuations of all three chromosome lengths are illustrated in Fig. 3a as well as the best-fit curves employing Timoshenko (5) and Euler-Bernoulli (7) theory. We employ a standard “fit” function in MATLAB™ that employs “Nonlinear Least Squares” to solve for (fit) the four unknown model parameters (*R*_1_, *R*_2_, *τ*_1_, and *τ*_2_) in (5) and the two unknown model parameters (*R* and *τ*) in (7) to the experimental data.

Notice that the experimental data deviate from the single time scale behavior (7), a feature most evident for the shortest chromosome (7 μm); see Fig. 3b and note log scale. For all lengths, the data clearly exhibit the two time scale behavior consistent with (5). Consequently, Timoshenko theory yields superior fits to the experimental data for all chromosome lengths (Fig. 3b–d) or averaged across all lengths (Fig. 3a). To quantify the degree of fit, we report the root mean squared error (RMSE) between the model fit to the experimental autocorrelation in Table 1; refer to column 4. The root mean squared error using Timoshenko theory remains less than that for the Euler-Bernoulli theory by a factor of 4 for the shortest length (Fig. 3b) to a factor of 2 (Fig. 3d) for the longest length. This trend confirms the expectation that shear deformation becomes increasingly important with the shorter filament lengths commonly found in the cell.

**Table 1:** The relaxation times and internal dissipation coefficients of three chromosomes modeled by Euler-Bernoulli (EB) theory and Timoshenko (T) theory.

Chromosome length, *L* (μm)	Dominant wavenumber, *q*_*dom*_ ≈ *π*/2*L* (μm^−1^)	Theory	RMSE	Relaxation time (s) from fitting (5) and (7) to experimental data	Internal dissipation coefficients (kg/(m.s)) at dominant wavenumber using (6) and (8)	Internal dissipation coefficients (kg/(m.s)) at large wavenumber limit using (16) and (17)
7	0.22	EB	0.052	*τ* = 0.64	${\eta ^{\prime} }_{b}=319$	${\eta ^{\prime} }_{b}=320$
		T	0.013	*τ*_1_ = 0.13, *τ*_2_ = 0.95	${\eta ^{\prime} }_{b}=66$, ${\eta ^{\prime} }_{s}=162$	${\eta ^{\prime} }_{b}=65$, ${\eta ^{\prime} }_{s}=162$
16.5	0.09	EB	0.039	*τ* = 0.66	${\eta ^{\prime} }_{b}=315\,$	${\eta ^{\prime} }_{b}=330\,$
		T	0.012	*τ*_1_ = 0.27, *τ*_2_ = 1.16	${\eta ^{\prime} }_{b}=119$, ${\eta ^{\prime} }_{s}=198$	${\eta ^{\prime} }_{b}=135$, ${\eta ^{\prime} }_{s}=198$
18.5	0.08	EB	0.030	*τ* = 0.87	${\eta ^{\prime} }_{b}=410$	${\eta ^{\prime} }_{b}=435$
		T	0.015	*τ*_1_ = 0.23, *τ*_2_ = 1.23	${\eta ^{\prime} }_{b}=93$, ${\eta ^{\prime} }_{s}=210$	${\eta ^{\prime} }_{b}=116$, ${\eta ^{\prime} }_{s}=210$
All three-lengths combined	—	EB	0.050	*τ* = 0.70	—	${\eta ^{\prime} }_{b}=350$
		T	0.031	*τ*_1_ = 0.22, *τ*_2_ = 1.13	—	${\eta ^{\prime} }_{b}=110$, ${\eta ^{\prime} }_{s}=192$

The root mean squared error (RMSE) represents the square root of the integral of the square of the difference between the experimentally measured autocorrelation and the associated theoretical fit.

The thermal fluctuations of the chromosomes are dominated by the smallest wavenumber bending mode, *q* ≈ *π*/2*L* for cantilevered chromosomes. We also report in Table 1 the dominant wavenumber for each of the three chromosome lengths and the associated relaxation times and internal dissipation coefficients as predicted by Timoshenko (5) and Euler-Bernoulli (7) theory. From (16) (and as illustrated in Fig. 2), the relaxation times in the large wavenumber limit are approximately ${\tau }_{1}\approx \frac{{\eta ^{\prime} }_{b}I}{B}$ for *q* > 0.06 μm^−1^ and ${\tau }_{2}\approx \frac{{\eta ^{\prime} }_{s}A}{\kappa S}$ for *q* > 0.007 μm^−1^, respectively. Since the dominant wavenumbers of the three chromosomes (*q*_*dom*_ = 0.22, 0.09, 0.08 μm^−1^) are all within the range of this limiting behavior (*q* > 0.06, 0.007 μm^−1^), we expect the internal viscosity coefficients ${\eta ^{\prime} }_{b}$ and ${\eta ^{\prime} }_{s}$ from (6) to be consistent with those of the large wavenumber limit (16) which are also reported in Table 1 (last column). The process of fitting two exponentials (5) to the experimental autocorrelations via nonlinear least squares yields two solutions for *τ*_1_ and *τ*_2_. For example, for the 7 μm length chromosome in Fig. 3, we obtain the two solutions:

(*a*) *τ*_1_ = 0.13, *τ*_2_ = 0.95

(*b*) *τ*_1_ = 0.95, *τ*_2_ = 0.13

Since the dominant wavenumber of the 7 μm length chromosome (*q*_*dom*_ = 0.22 μm^−1^) is in the large wavenumber limit, we expect the internal viscosity coefficients ${\eta ^{\prime} }_{b}$ and ${\eta ^{\prime} }_{s}$ from (6) to be consistent with those of the large wavenumber limit (16). Accordingly, the first solution (a) is accepted and the second solution (b) is rejected. This procedure leads to the relaxation times and the associated internal dissipation coefficients for bending and shear reported in Table 1 (and for all chromosome lengths).

The effective internal dissipation coefficient of mitotic chromosomes measured by dynamic force relaxation has been reported as *η*′ ≈ 100 kg/(m.s)^21^. Consequently, Poirier and Marko^12^ expected the bending relaxation time to be 0.3s. However, the estimated relaxation time based on Euler-Bernoulli theory (7) for the three chromosomes yields *τ* ≈ 0.7s^12^ and ${\eta ^{\prime} }_{b}\approx 350\,kg/({\rm{m}}.{\rm{s}})$; refer to Table 1 and Fig. 3a. By contrast, the relaxation time *τ*_1_ based on Timoshenko theory (5) yields *τ*_1_ ≈ 0.22s, which is significantly closer to the expected value. Consequently, the bending internal dissipation coefficient ${\eta ^{\prime} }_{b}\approx 110\,kg/({\rm{m}}.{\rm{s}})$ from Timoshenko theory (16) is also consistent with the experimental value *η*′ ≈ 100 kg/(m.s)^21^. Further, note that while the relaxation time calculated by Euler-Bernoulli theory (7) is close to the average of two relaxation times calculated by Timoshenko theory (5), the latter captures the two-stage (two-exponential) relaxation behavior evident in the experimental results of Fig. 3 for all chromosome lengths. Finally note that, as mentioned before, the effect of shear increases in the large wavenumber limit, specifically when *λ*/*r* < 10^14^, and this limit naturally arises in the spectrum of the thermal fluctuations of biofilaments.

### Effect of shear on external friction due to hydrodynamic drag

**Figure 4:**
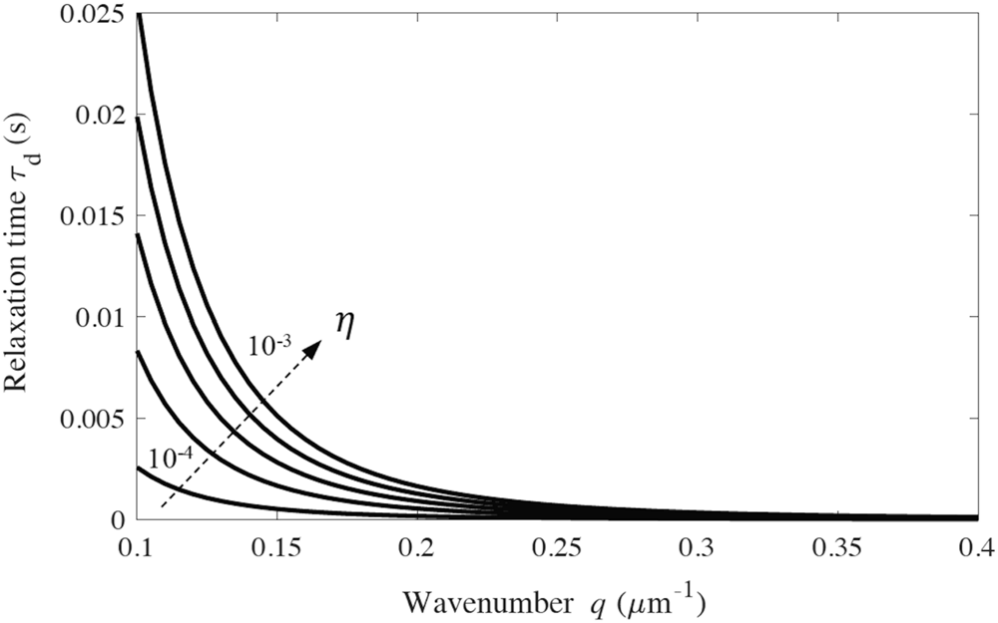
Relaxation time *τ*_*d*_ for the autocorrelation of transverse displacement of thermally fluctuating chromosomes with *E* = 500 Pa, *r* = 1 μm^12^, *G* = 227 Pa, ${\eta ^{\prime} }_{s}={\eta ^{\prime} }_{b}=0$, and *η* = 0.0001−0.001 kg/(m.s).

Figure 4 illustrates the predicted dependence of *τ*_*d*_ with wavenumber *q* over a wide range of *η* for (chromosomal) filaments. In the large wavenumber limit (*q* → ∞) in (13), *τ*_*d*_ → $\frac{\eta }{\kappa S{q}^{2}}\to 0$; see Fig. 4. In the small wavenumber limit (*q* → 0) in (13), *τ*_*d*_ → $\frac{\eta }{B{q}^{4}}$ which recovers the limiting behavior of Euler-Bernoulli theory (15). Thus, shear deformation can be ignored in the small wavenumber limit for fluctuating filaments having no internal friction. Also in this limit, the wavelength approaches infinity and the filament fluctuations reduce to rigid body motions.

### Experimental evidence for shear effect on hydrodynamic drag

**Figure 5:**
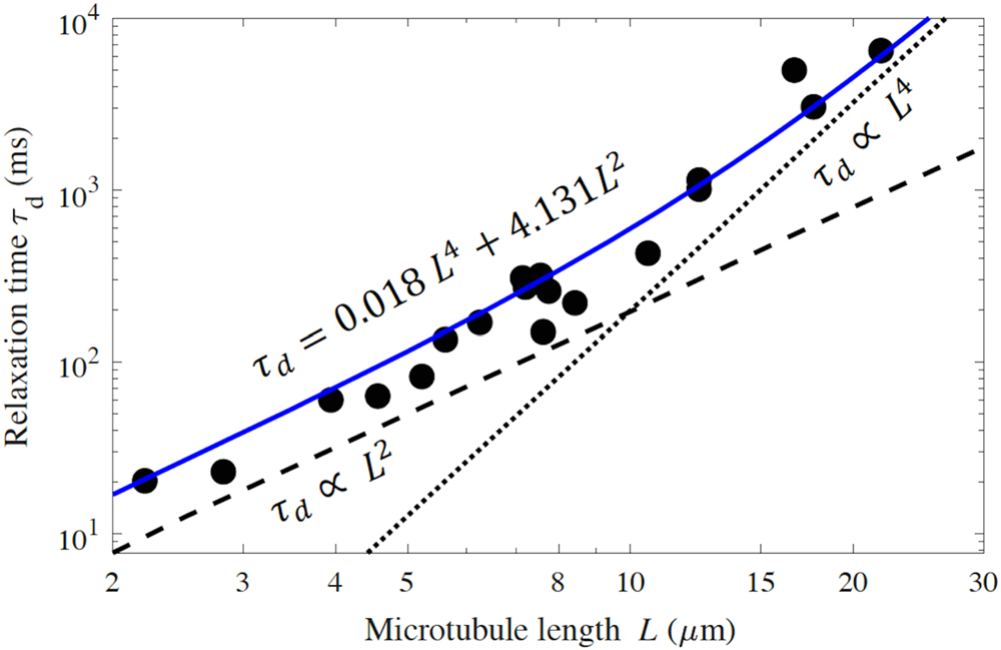
Relaxation time extracted from mean squared displacement of microtubules vs. microtubule length as measured by Taute *et al*.^13^ from thermal fluctuations of filaments. The fitted dotted-line with slope 4 and the dashed-line with slope 2 offered by Taute *et al*.^13^ reproduce the asymptotes of the Timoshenko theory (14) (blue curve) which remains a good approximation for all lengths.

In this second example, we consider the experiments of Taute *et al*.^13^ on thermal fluctuations of microtubules spanning lengths 2–30 μm. Figure 5 illustrates the experimentally determined relaxation time (for mean squared transverse displacement) as a function of microtubule length *L* for the fluctuations of the cantilevered microtubules. As described in^13^, Fig. 5 reveals that the relaxation time scales as *L*^4^ for microtubules longer than 10 μm. However, for microtubules shorter than 10 μm, the relaxation time scales as *L*^2^ instead, which deviates from Euler-Bernoulli theory (15) in which *η*′ = 0. Suspecting the influence of internal friction, Taute *et al*. employed (9) based on Euler-Bernoulli theory, however, the relaxation time (9) scales as *L*^4^ and *L*^0^ for large and small lengths, respectively. This mismatch between the experimental data and the theoretical models ((9) and (15)) for short-length filaments arises from neglecting the shear effect in the WLC model based on Euler-Bernoulli beam theory. The experimentally-observed scaling (*L*^4^ and *L*^2^ for large and small lengths, respectively) is instead revealed by (14) which captures the influence of shear deformation. We provide in Fig. 5 the best-fit curve to the experimental data using Timoshenko theory (14). Inspection reveals that the prediction based on Timoshenko theory remains a good fit at all lengths and, importantly, that it conforms to the asymptotic behaviors noted in the experiments at short lengths (*L*^2^–dependence) and long lengths (*L*^4^–dependence).

As mentioned above, shear deformation will ultimately become dominant for higher order modes, in particular, for modes that satisfy *λ*_*n*_/*r* < 10, where *n* is the mode number. For the clamped-free microtubules studied therein, the wavelengths are *λ*_*n*_ ≅ 2*L*/(2*n*−1)*π*. Accordingly, the above criterion leads to the following predictions of which modes will be dominated by shear as a function of microtubule length.

From Table 2, for the shorter length (2 *μm*) microtubules, shear will dominate bending for all modes *n* > 5 while still also playing an active role for modes *n* < 5 (though to a lesser degree as the mode number decreases). Of course, bending will dominate the modes of the longer length microtubules as expected. These conclusions are evident in Fig. 5, which considers the entire range 2 < *L* < 30 *μm*, and which illustrates that shear deformation is non-negligible for microtubules as *L* decreases. While this result confirms the importance of shear deformation for short microtubules, it also predicts that the dominant source of dissipation in this experiment derives from hydrodynamic effects rather than the internal friction effects as originally suspected in^13^.

**Table 2:** The mode numbers in which shear deformation becomes dominant as a function of microtubule length.

*L* = 2 *μm*	*L* = 10 *μm*	*L* = 30 *μm*
*n* > 5	*n* > 25	*n* > 77

## Conclusions

In this paper, we reveal the significant influence of shear deformation in the energy dissipation of thermally fluctuating biofilaments. In particular, the influence of shear becomes pronounced for short filaments (*L*/2*r* < 10) or for short wavelength fluctuations (*λ*/*r* < 10). We present a new model for the energy dissipation mechanisms in biofilaments deriving from both internal and external friction. The model, based on Timoshenko beam theory, explicitly accounts for the direct shear of filaments that is ignored in the worm-like chain model based on Euler-Bernoulli beam theory. The new model predicts that shear deformation leads to energy relaxation on two time scales associated with internal friction and on two length scales associated with external friction. With shear effects included, the model replicates experimental behaviors observed for thermally fluctuating chromosomes, with dissipation dominated by internal friction, as well as for thermally fluctuating microtubules, with dissipation dominated by hydrodynamic drag. The model is offered in an analytical form that can be readily employed in future studies of thermally fluctuating biofilaments.

## Electronic supplementary material

Supplementary Information
