# Supplementary material for: Shear Deformation Dissipates Energy in Biofilaments
Source: Sci Rep. 2018 Aug 3;8:11684. doi: 10.1038/s41598-018-29905-6 (PMC6076251; doi:10.1038/s41598-018-29905-6)
Supplement: Supplementary file 1 — Supplementary Information [file 41598_2018_29905_MOESM1_ESM.pdf]

Supplementary Information for
"Shear Deformation Dissipates Energy in Biofilaments"

Ameneh Maghsoodi, Noel Perkins
Department of Mechanical Engineering, University of Michigan, 2350 Hayward Street, Ann Arbor, MI 48109

Background on Timoshenko beam model for thermally fluctuating biofilaments

In this paper, we propose a theory for thermally fluctuating that captures both shear and bending deformation effects per Timoshenko beam theory[14]. The governing Equations (1-2) are deduced from Hamilton's Principle

$$\int_{t_1}^{t_2} \delta(-V + W_{th} - W_{ex} - W_{in})\mathrm{d}t = 0 \tag{S1}$$

in which the elastic energy V and work by random thermal noise n are

$$V = \int_0^L \frac{1}{2} B \left(\frac{\partial \varphi}{\partial x}\right)^2 \mathrm{d}x + \int_0^L \frac{1}{2} \kappa S \left(\frac{\partial u}{\partial x} - \varphi\right)^2 \mathrm{d}x \tag{S2}$$

$$W_{th} = \int_0^L n(x,t) u \mathrm{d}x \tag{S3}$$

Here, $\frac{\partial u}{\partial x}$ is the total rotation of the cross section of the biofilament due to bending and shear deformations and φ is the component due to bending alone. The quantities W_{ex} and W_{in} denote energy dissipated by external friction (hydrodynamic drag) and internal friction, respectively which are introduced in (S1) using Rayleigh dissipation functions. The rate of energy dissipation due to hydrodynamic drag is

$$\dot{E}_{ex} = \int_0^L \frac{1}{2} \eta \left(\frac{\partial u}{\partial t}\right)^2 \mathrm{d}x \tag{S4}$$

which, employing the Rayleigh dissipation function, results in

$$\int_{t_1}^{t_2} \delta W_{ex}\, dt = \int_{t_1}^{t_2} \frac{\partial \dot{E}_{ex}}{\partial \dot{u}}\, \delta u dt \tag{S5}$$

Similarly, the rate of energy dissipation due to internal friction is

$$\dot{E}_{in} = \int_0^L \frac{1}{2} \eta_b' I \left(\frac{\partial \dot{\varphi}}{\partial x}\right)^2 dx + \int_0^L \frac{1}{2} \eta_s' A \left(\frac{\partial \dot{u}}{\partial x} - \dot{\varphi}\right)^2 dx \tag{S6}$$

in which the first and second terms capture the rates of energy dissipation due to bending deformation and shear deformation, respectively. Note the structure of (S6) that is analogous to that of the elastic energy (S2). Employing the associated Rayleigh dissipation function results in

$$\int_{t_1}^{t_2} \delta W_{in}\,\mathrm{d}t = \int_{t_1}^{t_2} \left\{ \frac{\partial \dot{E}_{in}}{\partial\left(\frac{\partial \dot{\varphi}}{\partial x}\right)}\,\delta\left(\frac{\partial \varphi}{\partial x}\right) + \frac{\partial \dot{E}_{in}}{\partial\left(\frac{\partial \dot{u}}{\partial x} - \dot{\varphi}\right)}\,\delta\left(\frac{\partial u}{\partial x} - \varphi\right) \right\} \mathrm{d}t \tag{S7}$$

Substituting (S2-S7) into Hamilton's Principle (S1) and integrating by parts results in the following Langevin formulation ((1-2) in the paper)

$$B\frac{\partial^2 \varphi}{\partial x^2} + \kappa S\left(\frac{\partial u}{\partial x} - \varphi\right) + \eta_b' I \frac{\partial^3 \varphi}{\partial x^2 \partial t} + \eta_s' A\left(\frac{\partial^2 u}{\partial x \partial t} - \frac{\partial \varphi}{\partial t}\right) = 0 \tag{S8}$$

$$\kappa S\left(\frac{\partial \varphi}{\partial x} - \frac{\partial^2 u}{\partial x^2}\right) + \eta_s' A\left(\frac{\partial^2 \varphi}{\partial x \partial t} - \frac{\partial^3 u}{\partial x^2 \partial t}\right) + \eta \frac{\partial u}{\partial t} = n(x,t) \tag{S9}$$

Note that if the shear deformation effect is neglected, the total rotation of cross section is due to bending alone is described by the constraint $\frac{\partial u}{\partial x} = \varphi$. Upon employing this constraint, the Eqns. (S2) and (S6-S7) reduce, respectively, to

$$V = \int_0^L \frac{1}{2} B \left(\frac{\partial^2 u}{\partial x^2}\right)^2 \mathrm{d}x \tag{S10}$$

$$\dot{E}_{in} = \int_0^L \frac{1}{2} \eta_b' I \left(\frac{\partial^2 \dot{u}}{\partial x^2}\right)^2 dx \tag{S11}$$

$$\int_{t_1}^{t_2} \delta W_{in}\,\mathrm{d}t = \int_{t_1}^{t_2} \left\{ \frac{\partial \dot{E}_{in}}{\partial\left(\frac{\partial^2 \dot{u}}{\partial x^2}\right)}\,\delta\left(\frac{\partial^2 u}{\partial x^2}\right) \right\} \mathrm{d}t \tag{S12}$$

In this limit, Timoshenko theory reduces to the Euler-Bernoulli theory employed in WLC model[12]. Substitution of (S3-S5) and (S10-S11) into (S1) yields

$$B\frac{\partial^4 u}{\partial s^4} + \eta\frac{\partial u}{\partial t} + \eta_b' I \frac{\partial}{\partial t}\frac{\partial^4 u}{\partial x^4} = n(s,t) \tag{S13}$$

as expected.

If the internal friction is neglected ($\eta_s' = \eta_b' = 0$), equations (S8-S9) reduce to ((10-11) in the paper)

$$B\frac{\partial^2\varphi}{\partial x^2}+\kappa S\left(\frac{\partial u}{\partial x}-\varphi\right)=0 \tag{S14}$$

$$\kappa S\left(\frac{\partial\varphi}{\partial x}-\frac{\partial^2 u}{\partial x^2}\right)+\eta\frac{\partial u}{\partial t}=n(x,t) \tag{S15}$$

Derivation of relaxation time constants

The Fourier transform of (S8) and (S9) with respect to space and time yields

$$\Phi_{q\omega}\big((-Bq^2-\kappa S)+i(\eta_b' I\omega q^2+\eta_s' A\omega)\big)+U_{q\omega}(\eta_s' A\omega q+i\kappa Sq)=0 \tag{S16}$$

$$\Phi_{q\omega}(\eta_s' A\omega q+i\kappa Sq)+U_{q\omega}\big(\kappa Sq^2+i(-\eta_s' A\omega q^2-\eta\omega)\big)=N_{q\omega} \tag{S17}$$

in which ω and q denote the frequency and wavenumber of propagating waves, respectively and $U_{q\omega}$, $\Phi_{q\omega}$, and $N_{q\omega}$ denote the (double) Fourier transforms of the transverse displacement u, the rotation φ, and the random thermal noise n, respectively. Solution of $U_{q\omega}$ yields

$$U_{q\omega}=\frac{\mathrm{A}+i\mathrm{B}}{\mathrm{C}+i\mathrm{D}}N_{q\omega} \tag{S18}$$

with

$$\mathrm{A}=Bq^2+\kappa S \tag{S19}$$

$$\mathrm{B}=-\omega(\eta_b' Iq^2+\eta_s' A)$$

$$\mathrm{C}=(\eta_s' A\omega q)^2-(\kappa Sq)^2+\kappa Sq^2(Bq^2+\kappa S)-(\eta_s' A\omega q^2+\eta\omega)(\eta_b' I\omega q^2+\eta_s' A\omega)$$

$$\mathrm{D}=2(\eta_s' A\omega q)(\kappa Sq)-\kappa Sq^2(\eta_b' I\omega q^2+\eta_s' A\omega)-(\eta_s' A\omega q^2+\eta\omega)(Bq^2+\kappa S)$$

The power spectral density for u follows from

$$\langle {U_{q\omega}}^2\rangle=\frac{\mathrm{A}^2+B^2}{\mathrm{M}\omega^4+\mathrm{N}\omega^2+\mathrm{P}}\langle {N_{q\omega}}^2\rangle \tag{S20}$$

with

$$\mathrm{M}=(\eta_s'\eta_b' AIq^4+\eta_b'\eta Iq^2+\eta_s'\eta A)^2$$

$$\begin{aligned}\mathrm{N}=&\ q^8((\eta_s' AB)^2+(\eta_b' I\kappa S)^2)+q^6(2\eta_s'\eta AB^2)\\&+q^4(2\eta\eta_b' I(\kappa S)^2+(B\eta)^2)+q^2(2\kappa SB\eta^2)+(\kappa S\eta)^2\end{aligned} \tag{S21}$$

$$\mathrm{P}=q^8(\kappa SB)^2$$

The time autocorrelation function $\mathcal{R}(T)$ for u follows from the inverse Fourier transform of (S20) with respect to ω in which one assumes ideal (white) random thermal noise which yields ((5-6) in the paper)

$$\mathcal{R}(T) = R_1 \exp\left(\frac{-T}{\tau_1}\right) + R_2 \exp\left(\frac{-T}{\tau_2}\right) \tag{S22}$$

$$\tau_1 = \sqrt{\frac{2\mathrm{M}}{\mathrm{N}+\sqrt{\mathrm{N}^2-4\mathrm{MP}}}}, \quad \tau_2 = \sqrt{\frac{2\mathrm{M}}{\mathrm{N}-\sqrt{\mathrm{N}^2-4\mathrm{MP}}}} \tag{S23}$$

with

$$R_1 = \frac{2k_B TL(Bq^2+\kappa S)}{\kappa SBq^4}\frac{R_3}{R_3+R_4} \tag{S24}$$

$$R_2 = \frac{2k_B TL(Bq^2+\kappa S)}{\kappa SBq^4}\frac{R_4}{R_3+R_4} \tag{S25}$$

$$R_3 = \frac{(\eta_b' Iq^2+\eta_s' A)^2 - {\tau_1}^2(Bq^2+\kappa S)^2}{2L({\tau_2}^2-{\tau_1}^2)}\tau_1{\tau_2}^2 \tag{S26}$$

$$R_4 = \frac{{\tau_2}^2(Bq^2+\kappa S)^2 - (\eta_b' Iq^2+\eta_s' A)^2}{2L({\tau_2}^2-{\tau_1}^2)}\tau_2{\tau_1}^2 \tag{S27}$$

Note that $\langle {N_{q\omega}}^2 \rangle$ is a function of wavenumber q only. To find thermal noise, we first rewrite the elastic energy (S2) in terms of wavenumber q

$$V = \frac{B}{2L}\sum_q {U_q}^2\, q^4 \left(\frac{\kappa S}{\kappa S+Bq^2}\right)^2 + \frac{\kappa S}{2L}\sum_q {U_q}^2\, q^4 \left(\frac{Bq}{\kappa S+Bq^2}\right)^2 \tag{S28}$$

From equipartition theorem and (S28),

$$\langle {U_q}^2 \rangle = \frac{2k_B TL(Bq^2+\kappa S)}{\kappa SBq^4} \tag{S29}$$

Transforming (S20) from frequency domain to time domain must recover (S29) at equal time. In doing so, the thermal noise correlation in wavenumber and time domains is

$$\langle {N_q}^2 \rangle = \frac{2k_B TL(Bq^2+\kappa S)}{\kappa SBq^4}\frac{1}{R_3+R_4} \tag{S30}$$

Equations (S20-S30) confirm that the thermal noise is related to the two internal dissipation coefficients η_s' and η_b', external dissipation coefficient η, and to absolute temperature T.

If the internal friction is neglected, i.e., $\eta_s' = \eta_b' = 0$, the Fourier transforms of (S14) and (S15) with respect to space and time yield

$$\Phi_{q\omega}(-Bq^2 - \kappa S) + U_{q\omega}(i\kappa Sq) = 0 \tag{S31}$$

$$\Phi_{q\omega}(i\kappa Sq) + U_{q\omega}\big(\kappa Sq^2 + i(-\eta\omega)\big) = N_{q\omega} \tag{S32}$$

Then, the power spectral density for u follows from

$$\langle {U_{q\omega}}^2 \rangle = \frac{(Bq^2 + \kappa S)^2}{(\kappa S\eta + B\eta q^2)^2\omega^2 + (\kappa SBq^4)^2} \langle {N_{q\omega}}^2 \rangle \tag{S33}$$

The inverse Fourier transform of (S33) with respect to ω yields the associated autocorrelation $\mathcal{R}(T)$ and relaxation time τ_d become ((12-13) in the paper)

$$\mathcal{R}(T) = R_d \exp\left(\frac{-T}{\tau_d}\right) \tag{S34}$$

$$\tau_d = \frac{\kappa S\eta + B\eta q^2}{\kappa SBq^4} \tag{S35}$$

$$R_d = \frac{2k_B TL(Bq^2 + \kappa S)}{\kappa SBq^4} \tag{S36}$$

Transforming (S33) to equal times must recover (S29). In this case, the thermal noise correlation in wavenumber and time domains is

$$\langle {N_q}^2 \rangle = 4k_B TL\,\eta \tag{S37}$$

Equation (S37) confirms that the amplitude of thermal noise is proportional to the absolute temperature T and external viscosity coefficient η.
